# Structural and Functional Characterization of a Multifunctional Alanine-Rich Peptide Analogue from *Pleuronectes americanus*

**DOI:** 10.1371/journal.pone.0047047

**Published:** 2012-10-08

**Authors:** Ludovico Migliolo, Osmar N. Silva, Paula A. Silva, Maysa P. Costa, Carolina R. Costa, Diego O. Nolasco, João A. R. G. Barbosa, Maria R. R. Silva, Marcelo P. Bemquerer, Lidia M. P. Lima, Maria T. V. Romanos, Sonia M. Freitas, Beatriz S. Magalhães, Octavio L. Franco

**Affiliations:** 1 Centro de Análises Proteômicas e Bioquímicas-Programa de Pós-Graduação em Ciências Genômicas e Biotecnologia, Universidade Católica de Brasília, Brasília, Distrito Federal, Brazil; 2 Programa de Pós-Graduação em Genética e Biotecnologia, Universidade Federal de Juiz de Fora, Juiz de Fora, Minas Gerais, Brazil; 3 Laboratório de Biofísica-Departamento de Biologia Celular, Universidade de Brasília, Brasília, Distrito Federal, Brazil; 4 Laboratório de Sîntese de Peptídeos, EMBRAPA Recursos Genéticos e Biotecnologia, Brasília, Brazil; 5 Instituto de Patologia Tropical e Saúde Pública, Universidade Federal de Goiás, Goiânia, Goiás, Brazil; 6 Departamento de Virologia-Instituto de Microbiologia Paulo Góes, Universidade Federal do Rio de Janeiro, Rio de Janeiro, Rio de Janeiro, Brazil; Russian Academy of Sciences, Institute for Biological Instrumentation, Russian Federation

## Abstract

Recently, defense peptides that are able to act against several targets have been characterized. The present work focuses on structural and functional evaluation of the peptide analogue *Pa-*MAP, previously isolated as an antifreeze peptide from *Pleuronectes americanus*. *Pa-*MAP showed activities against different targets such as tumoral cells in culture (CACO-2, MCF-7 and HCT-116), bacteria (*Escherichia coli* ATCC 8739 and *Staphylococcus aureus* ATCC 25923), viruses (HSV-1 and HSV-2) and fungi (*Candida parapsilosis* ATCC 22019, *Trichophyton mentagrophytes* (28d&E) and *T. rubrum* (327)). This peptide did not show toxicity against mammalian cells such as erythrocytes, Vero and RAW 264.7 cells. Molecular mechanism of action was related to hydrophobic residues, since only the terminal amino group is charged at pH 7 as confirmed by potentiometric titration. In order to shed some light on its structure-function relations, *in vitro* and *in silico* assays were carried out using circular dichroism and molecular dynamics. Furthermore, *Pa*-MAP showed partial unfolding of the peptide changes in a wide pH (3 to 11) and temperature (25 to 95°C) ranges, although it might not reach complete unfolding at 95°C, suggesting a high conformational stability. This peptide also showed a conformational transition with a partial α-helical fold in water and a full α-helical core in SDS and TFE environments. These results were corroborated by spectral data measured at 222 nm and by 50 ns dynamic simulation. In conclusion, data reported here show that *Pa-*MAP is a potential candidate for drug design against pathogenic microorganisms due to its structural stability and wide activity against a range of targets.

## Introduction

Recently, advances in molecular biology have explained the complexity of life while retaining the simplicity of biology’s central dogma. Indeed, in the last few years many researchers have demonstrated that proteins and peptides possess a remarkable ability to adapt to the environment and develop a number of functions under specific conditions. In this context, protein and peptide promiscuity, in which multiple functions may be associated with a single structure in different environments, has been gaining attention in various research fields, including immunology, biochemistry and chemistry [1]. In fact, multiple functions seem to be an essential strategy of peptide evolution, facilitating the divergence of novel functions within accessible folds, and probably leading finally to the evolution of entirely new peptide functions.

In line with this view, the discovery of novel multifunctional peptides has been extremely important for understanding action mechanisms, behavior in different environmental parameters (pH and temperature, for example) and folding of several peptides. These bioactive molecules can be obtained from a wide variety of sources, including microorganisms, plants, amphibians, insects, mollusks and fish [2]. In Teleostei marine polar fish, antifreeze peptides (AFPs) are commonly secreted into the blood and various tissues, reaching a concentration of 10 to 40 mg.mL^−1^, depending on sub-zero temperature [3], [4]. The type I AFP family is commonly characterized from winter flounder (*Pleuronectes americanus*) by two peptides that present lower molecular masses, named HPLC-6 (4000 Da) and HPLC-8 (3300 Da) [5]. The secondary structure of peptides from this class consists of an alanine-rich α-helix composed of eleven amino acid residues with three imperfect motif repetitions (X_10_T, where *X* is any amino acid residue and *T* is threonine) [6]. A comparison between AFPs and antimicrobial peptides (AMPs) has revealed similar structural and physical-chemical properties, including hydrophobic ratio (45 to 55% hydrophobicity), polypeptide chain length (twenty to thirty amino acid residues), hydrophobic moment and a specific amino acid composition (mainly threonine, leucine, histidine, lysine, arginine, aspartic acid and alanine) [7]. These characteristics probably reflect the fact that many of these peptides may be capable of interacting with and disrupting target cell membranes [8]. Considering these shared characteristics, the key question is: Do these fish-sourced peptides have a single function? It seems that they do not, since the concept of peptides with multiple functions has been commonly observed in different organisms, representing savings in energy for organisms that express a single gene with multiple activities [1], [9].

Experiments in the 1990s suggested that both AFPs and AMPs might interact with biological membranes. However, the composition of cell membrane phospholipids and cholesterol is a determining factor in achieving cryopreservation or causing damage [10]. A decade later, Tomczak and Crowe proposed that the mechanisms of membrane stabilization and protection caused by type I AFP could be related and might be oriented by peptide insertion into the membrane [11]. Recently, studies demonstrated that stabilization of antifreeze peptides in the 1,2-dimyristoyl-sn-glycero-3-phosphocholine (DMPC) model membrane using short segments of type I AFP could occur due to hydrophobic interactions of peptide with membrane core [12]. Additionally, the understanding of peptide-membrane interaction may allow the discovery and design of new biopharmaceuticals with high efficiency in controlling pathogenic microorganisms. The application of multiple active peptides (MAPs) in the control of high frequency infections caused by viruses, bacteria and fungi could open new frontiers in the search to identify alternative biocides whose novel mode of action may slow down the alarming trend toward resistance [13].

Multiple antifreeze peptide motifs from *P. americanus* (winter flounder) were obtained through NCBI database data mining. The antifreeze peptide was synthetically constructed with few differences of original peptide that was firstly expressed in bacterial heterologous system [14]. Here the peptide named *Pa*-MAP show two modifications, the absence of first amino acid residue methionine (initiation codon), unnecessary since we utilized chemical synthesis for peptide production and also showed a amidated C-termini, in order to improve peptide protection against medium degradation. This study uses chemical solid-phase synthesis with Fmoc strategy to produce a peptide with two 11-amino acid imperfect repeats. *Pa-*MAP was evaluated for its action toward multiple human-pathogenic microorganisms. Moreover, its secondary structure was also studied by circular dichroism (CD) and corroborated by further *in silico* comparative modeling and molecular dynamics (MD).

## Experimental Section

### Sequence Target for Studies of Multiple Activities

The primary sequence selected from a polar fish for multifunctional analysis consists of a characterized antifreeze peptide sequence from *Pleuronectes americanus* named HPLC-8. The synthesized peptide was based on the HPLC-8 antifreeze motif with two imperfect long repeats of eleven amino acid residues as described previously by Holmberg and coworkers [14]. The NCBI protein database [15] was used to acquire the antifreeze sequence (number AAC60714), BioEdit [16] was used to analyze the physical-chemical parameter of the template and ClustalW [17] was used for comparison of sequences.

### Solid-phase Peptide Synthesis

The peptide was synthesized by the stepwise solid-phase method using the N-9-ﬂuorenylmethyloxycarbonyl (Fmoc) strategy with a Rink amide resin (0.52 mmol.g^−1^) [18]. Side chain protecting groups were t-butyl for threonine and (triphenyl)methyl for histidine. Couplings were performed with 1,3-diisopropylcarbodiimide/1-hydroxybenzotriazole (DIC/HOBt) in N,N-dimethylformamide (DMF) for 60 to 120 min. Fmoc deprotections (15 min, twice) were conducted with 4-methylpiperidine:DMF solution (1∶4; by volume). Cleavage from the resin and final deprotection of side chains were performed with triﬂuoroacetic acid (TFA):water:1,2-ethanedithiol (EDT): triisopropylsilane (TIS), 94.0∶2.5∶2.5∶1.0, by volume, at room temperature for 90 min. After this, the crude product was precipitated with cold diisopropyl ether, collected by filtration and solubilized in 200 mL aqueous acetonitrile at 50% (by volume). The extracted peptide was twice freeze-dried for purification. Amino acid derivatives and other reagents for the solid-phase peptide synthesis were from Merck-NovaBiochem (Whitehouse Station, NJ) or from Peptides International (Louisville, KY) or from Sigma-Aldrich (St Louis. MO).

### Peptide Purification

The crude peptide was solubilized in 0.1% triﬂuoroacetic acid (TFA) aqueous solution and filtered with a Millex filter 0.22 µm 25 mm (Millipore-Merck, Billerica, MA). The crude extract was submitted to semi-preparative reverse phase high-performance liquid chromatography (RP-HPLC), C18 NST, 5 µm, 250 mm×10 mm, using the following mobile phase conditions: H_2_O:ACN:TFA (95∶05:0.1, v:v:v) for 5 min, then a linear gradient to H_2_O:ACN:TFA (05∶95:0.1, v:v:v) for 60 min at a flow rate of 2.5 mL.min^−1^. The experiments were conducted at room temperature and monitored at 216 nm. Fractions were manually collected and lyophilized. The synthetic peptide concentrations for all *in vitro* experiments were determined by using the measurement of absorbance at 205, 215 and 225 nm, as described by Murphy and Kies [19].

### Mass Spectrometry Analyses

*Pa-*MAP molecular mass was determined by using matrix-assisted laser desorption/ionization time of flight mass spectrometry (MALDI-ToF MS/MS) analysis on UltraFlex III, Bruker Daltonics, Billerica, MA. Purified peptide was dissolved in a minimum volume of water that was mixed with an α-cyano-4-hydroxycinnamic acid saturated matrix solution (1∶3, v:v), spotted onto a MALDI target plate and dried at room temperature for 5 min. The α-cyano-4-hydroxycinnamic acid matrix solution was prepared at 50 mM in H_2_O:ACN:TFA (50∶50:0.3, v:v:v). Peptide monoisotopic mass was obtained in the reﬂector mode with external calibration, using the Peptide Calibration Standard II for mass spectrometry (up to 4,000 Da mass range, Bruker Daltonics, Billerica, MA).

### Potentiometric Titration

Aiming to identify the contribution of His1 amino acid residue, a potentiometric titration was realized according Crouch and coworkers [20]. This experiment demonstrated that the His^1^ was not protonated and therefore did not contributed with positive charge (Figure S1). The potentiometric titration curve for *Pa*-MAP was constructed using a DM –21 (Digimed, São Paulo, Brazil) pH meter coupled to a glass electrode combined with a thermo-compensating apparatus. A solution containing 5 mL of synthetic peptide in the concentration of 0.27 mM was solubilized in distilled water and acidified with a solution of hydrochloric acid, 1 mM until pH 3. Subsequently, the sample was titrated with the increasing volumes of 1 mM sodium hydroxide, until pH 9. Obtained pH values were registered as function of volume of added KOH.

### Hemolytic Assays

The hemolytic activity of *Pa-*MAP was determined by using fresh human erythrocytes from healthy donors. Human heparine-blood was obtained from the Hospital of the Catholic University of Brasilia cell collection and stored at 4°C. Collection was obtained with written informed consent. Blood was centrifuged and the erythrocytes were washed three times with 50 mM phosphate buffer, pH 7.4. The peptide solution was added to the erythrocyte suspension (1%, by volume), at a final concentration ranging from 2 to 115 µM in a final volume of 100 µL. Samples were incubated at room temperature for 60 min. Hemoglobin release was monitored by measuring the supernatant absorbance at 540 nm. Zero hemolysis control (blank) was determined with erythrocytes suspended in the presence of 50 mM phosphate buffer, pH 7.4, and for positive control (100% of erythrocyte lyses); an aqueous solution of 1% (by volume) triton X-100 dissolved in distilled water was used instead of the peptide solution. This study was approved by the Animal Use Committee at the Institute of Biological Sciences, University of Brasilia. The hemolytic assays were performed in triplicate.

### Cytotoxicity Assay

In order to determine the maximum non-toxic concentrations (MNTC) of the peptide *Pa*-MAP, several concentrations of peptide (200, 100, 50, 25, 12.5, 6.25 and 3.15 µg.mL^−1^) were assayed with conﬂuent RAW 264.7 (mouse leukemic monocyte macrophage) cell line and incubated at 37°C in a 5% CO_2_ atmosphere for 48 h. After incubation, the cells were examined using an inverted optical microscope (Leitz) aiming to evaluate morphological alterations. Cellular viability was further evaluated by the neutral red dye-uptake method [21]. Cells were incubated in the presence of 0.01% (by weight) neutral red solution for 3 h at 37°C in a 5% CO_2_ atmosphere. Then the medium was removed and the cells were fixed with 4% formalin in phosphate-buffered saline, pH 7.2. The dye, incorporated by the viable cells, was eluted by using a mixture of methanol:acetic acid:water (50∶1:49, v:v:v), and the dye uptake was determined by measuring the absorbance at 490 nm in an automatic spectrophotometer (ELx800 TM-Bio-TeK Instruments, Inc.). The 50% cytotoxic concentration (CC_50_) was defined as the concentration that caused 50% reduction in dye uptake. The cytotoxic assays were performed in triplicate.

### Assays for Cancer Cells in Culture

CACO-2 (heterogeneous human epithelial colorectal adenocarcinoma), MCF-7 (human breast cancer) and HCT-116 (human colorectal carcinoma) cells were acquired from the Cell Bank in Rio de Janeiro (CR108). The cells were cultured in Dulbecco’s Modified Eagle Medium (DMEM Gibco), supplemented with 10% fetal serum bovine, penicillin (100 U.mL^−1^) and streptomycin (100 µg.mL^−1^), and maintained at 37°C in 5% of CO_2_ atmosphere (Invitrogen, Burlington, ON, Canada). Evaluation of the peptide against the tumor cells described above was assayed in two-fold dilutions (from 512 to 4 µg.mL^−1^). An MTT (3-(4,5-dimethylthiazol-2yl)-2,5-diphenyltetrazolium bromide) cytotoxicity test was used at 1 mg.mL^- 1^ to evaluate the cell viability after cells had been incubated with samples for periods of 24, 48 and 72 h. The ED_50_ values were calculated as the amount of *Pa-*MAP required to produce an inhibitory effect on the development in half of the population of cancer cells cultured *in vitro*. The cell culture assays were performed in triplicate.

### Antifungal Tests

The minimum inhibitory concentrations (MICs) of *Pa*-MAP were determined by using the broth microdilution method according to the Clinical and Laboratory Standards Institute (CLSI) M27-S3 [22] with Roswell Park Memorial Institute (RPMI) 1640 medium. Stock solutions of peptide were dissolved in RPMI 1640 medium. The final concentrations ranged from 0.25 to 264 µg.mL^−1^. Briefly, a standard inoculum of clinically isolated (28d&E) *Candida parapsilosis* ATCC 22019, *Trichophyton mentagrophytes var. mentagrophytes* and clinically isolated (327) *T. rubrum* was initially produced. The cell density was adjusted by turbidity measurements (at 530 nm wavelength) to yield a fungal stock of 1 × 10^6^ cfu per mL. Further dilutions were made with RPMI 1640 medium, resulting in a final inoculum of approximately 0.5 × 10^3^ to 2.5 × 10^3^ cells.mL^−1^. Next, 100 µL of the fungal suspension was incubated at 35°C and 100 µL of the *Pa-*MAP was placed in the wells of the microdilution tray. End points were visually read after 48 h for *C. parapsilosis* and 96 h for *T. mentagrophytes var. mentagrophytes* and *T. rubrum*. The MIC of *Pa-*MAP was considered as the lowest concentration that caused a complete growth inhibition (100%) when compared to control tube growth. Each antifungal test was carried out in triplicate.

### Antibacterial Tests

*Escherichia coli* ATCC 8739 and *Staphylococcus aureus* ATCC 25923 were used for antimicrobial assays. The bacterial species were cultured in 1.0 mL LB broth for 2 h, at 37°C in accordance with guidelines from the CLSI, 2009. The synthetic peptide was incubated with 5×10^6^ CFU.mL^−1^ for each bacterial species for 4 h, at 37°C. The negative and positive assay controls were bacteria in LB medium and in several dilutions of chloramphenicol, respectively. Bacterial growth was measured at 595 nm, every hour within the period of incubation, carried out according to protocols described by the National Committee for Clinical Laboratory Standards (NCLS) guidelines. All antibacterial experiments were carried out in triplicate. In addition, to determine the MIC, the peptide *Pa-*MAP was serially diluted from 256 to 2 µg.mL^−1^ in LB medium. The MIC was determined as the lowest concentration that caused complete growth inhibition (100%) in comparison to the negative control. In each well of a 96-well polypropylene plate, 100 µL of each dilution (medium + peptide) and 10 µL of cell suspension of bacteria were added (approximately 5×10^6^ CFU of bacteria). The plates were incubated for 12 h at 37°C. During this period the absorbance was measured in a plate reader (Bio-Rad 680 Microplate Reader) at 595 nm every 30 min.

### Cells and Viruses

Vero cells (African green monkey kidney cells) were grown in Eagle’s minimum essential medium (Eagle MEM) supplemented with 10% (by volume) fetal bovine serum, L-glutamine (0.03 mg.mL^−1^), garamycin (50 µg.mL^−1^), amphotericin B (2.5 mg.mL^−1^), NaHCO_3_ (0.25%) and 4-(2-hydroxyethyl)-1-piperazineethanesulfonic acid, HEPES (10 mM). Cell cultures were prepared in 96-well microtiter plates (Falcon Plastics, Oxnard, CA, USA) and incubated at 37°C in a 5% CO_2_ atmosphere. HSV-1 was isolated from a typical lip lesion and HSV-2 from a typical genital lesion in the Virology Department of the Universidade Federal do Rio de Janeiro (UFRJ), Brazil. Viruses were typed by polymerase chain reaction (PCR) using specific primers for identification [23].

### Antiviral Activity Assay

The antiviral activity of peptide *Pa-*MAP was evaluated by titer reduction. The virus titers were calculated using the statistical method and expressed as 50% tissue culture infective dose (TCID_50_) per mL [24]. Vero cell monolayers were treated with the peptide from 256 to 2 µg.mL^−1^ at the MNTC and 100 TCID_50_.mL^−1^ of HSV-1 or HSV-2 suspensions were added to treated and untreated cell cultures and incubated at 37°C for 48 h in a 5% CO_2_ atmosphere. After incubation, the supernatant was collected and virus titers in treated and untreated cells were determined. The antiviral activity was expressed as the percentage inhibition (PI) using antilogarithmic TCID_50_ values as follows: PI = [1 - (antilogarithmic test value/antilogarithmic control value)]×100. Test values consist of the number of viral particles produced in the presence of *Pa*-MAP and control value is the number of viral particle in the negative control without *Pa*-MAP. This formula was utilized to determine the viral inhibition percentage in according with Simões and coworkers [25]. The dose-response curve was established starting from the MNTC, and the 50% effective dose (ED_50_) was defined as the dose required to achieve 50% protection against virus-induced cytopathic effects [26]. The selectivity index (SI) was determined as the ratio of CC_50_ to ED_50_, and 2-amino-9-(2-hydroxyethoxymethyl)-3H-purin-6-one or Acyclovir (Sigma Chemical Company, St Louis) was used as standard compound. Each antiviral assay was performed in triplicate.

### Circular Dichroism Spectroscopy

Circular dichroism (CD) measurements were carried out on a JASCO J-815 spectropolarimeter (Easton, MD), equipped with a Peltier-type temperature controller, and a thermostable cell holder, interfaced with a thermostatic bath. Spectra were recorded in 0.1 cm path length quartz cells at a peptide concentration range of 0.05−0.5 mg.mL^−1^ in 2 mM Na-acetate buffer at pH 3.0, 2 mM Na-acetate buffer at pH 4.0, 2 mM Na-acetate buffer at pH 5.5, deionized water (Milli-Q), 2 mM Tris-HCl buffer at pH 7.0, 2 mM ammonium bicarbonate buffer at pH 8.5, 2 mM glycine-NaOH buffer at pH 10.0 and 2 mM glycine-NaOH buffer at pH 11.0. Four consecutive scans were accumulated and the average spectra stored. Thermal denaturation experiments were performed by increasing the temperature from 25 to 95°C, allowing temperature equilibration for 5 min before recording each spectrum. *Pa-*MAP analysis in the presence of sodium dodecyl sulfate (SDS) and 2,2,2-trifluoroethanol (TFE) were performed in the same quartz cell with a 0.1 cm path length at 20°C. The spectra were recorded between 190 and 260 nm at a scan speed of 50 nm.min^−1^ and six scans were performed per sample. The spectra were recorded in three average environments: distilled water, 28 mM SDS micelles, and 50% (by volume) TFE in water. The observed ellipticity was converted into the mean residue ellipticity [θ] based on a mean molecular mass per residue of 115 Da. The data were corrected for the baseline contribution of the buffer and the observed ellipticities at 222 nm were recorded. The α-helical content of the various peptides was calculated from mean residual ellipticity at 222 nm ([θ]222) using the following equation: fH = [θ]222/[−40,000(1−2.5/n)], where *fH* and *n* represent the α-helical content and the number of peptide bonds, respectively [27].

### In Silico Analyses and Molecular Modeling

The three-dimensional model for *Pa-*MAP was constructed based on the structure 1jb5 of the PDB, which presented 62% of identity between the primary sequences. Fifty theoretical three-dimensional peptide structures were constructed by Modeller v.9.8 [28] using the template. The final model was chosen as the best evaluated one using PROSA II [29] to analyze packing and solvent exposure characteristics and PROCHECK for additional analysis of stereochemical quality. In addition, RMSD value was calculated by overlapping the Cα traces and backbones onto the template structure with the 3DSS program aiming to identify and validate the best generated model. A small RMSD value reflects in a model with lower energy and consequently an enhanced structural stability [30]. The peptide structures were visualized and analyzed on Delano Scientific’s PYMOL - http://pymol.sourceforge.net/ [31]. The electrostatic surface was calculated with the ABPS tool [32]. The grand average of hydropathicity, known as GRAVY, was calculated using ProtParam software [33].

### Molecular Dynamics Simulation

The molecular dynamics simulations for the *Pa*-MAP model were carried out in two steps. The first step took place in water and the second one in TFE at 25 and 50% (by volume), similar to the in vitro analysis. The forcefield utilized was GROMOS96 43A1 and analyses were performed using the computational package GROMACS v.4. [34]. The dynamics had the best tridimensional model of Pa-MAP as initial structure, which was immersed in 7,897 water molecules in a cube box with sides measuring 6.22 nm. Sodium ions were also inserted to neutralize the charge of the system. A second step for TFE simulation at 50% was carried out with 1,892 and 2,023 water molecules and 459 and 354 TFE molecules in a cube box with sides measuring 4.78 nm for 25 and 50% of TFE, respectively. The *Pa*-MAP C-terminal was modified with an amide group for both steps [35]. Geometry of water and water/TFE 25 and 50% molecules was constrained using the SETTLE algorithm [36]. All atom bond lengths were linked by the LINCS algorithm [37]. Electrostatic corrections were made by algorithm Particle Mesh Ewald (PME) with a radius cut-off of 1.4 nm in order to minimize the computational simulation time. The same radius cut off was also used for van der Waals interactions. The list of neighbors of each atom was updated every 10 simulation steps of 2 fs each. A conjugate gradient (2 ns) and the steepest descent algorithms (2 ns) were implemented for energy minimization. After that, the system underwent a normalization of pressure and temperature, using the integrator stochastic dynamics (SD), 2 ns each. The system with minimized energy and balanced temperature and pressure was carried out using a step of position restraint, using the integrator Molecular Dynamics (MD), for 2 ns. The simulations were carried out at 20°C in silico, aiming to understand the structural conformation of the peptide in the presence of different environments. The total time of Pa-MAP simulation was 50 ns. The values obtained for radius of gyration, root mean square deviation and accessibility area represented the peptide flexibility. These values were obtained with linear regression are delta modules, encountered between the beginning and final simulation. This procedure was performed in order to shed some light over peptide stability. The linear regressions observed for the peptide in water were: y = 1.0927−6.9214e−06 * x; y = 0.52406+1.215e−05 * x and y = 15.195−4.1696e−05 * x for Rg, RMSD and Area. On the other hand, in TFE, the linear regressions observed were: y = 0.42061+2.0657e−07 * x; y = 1.2387 - 8.8112e−09 * x and y = 15.956 - 4.951e−07 * x for Rg, RMSD and Area, respectively.

## Results

### Design, Syntheses and Purification of Pa-MAP

**Figure 1:**
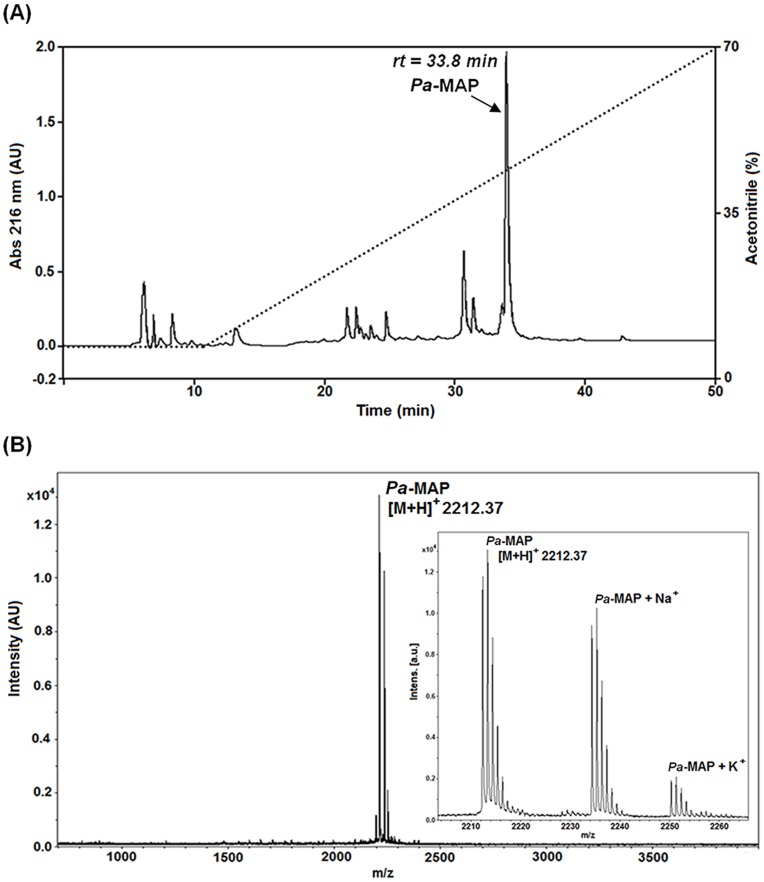
Purification profile of *Pa-*MAP synthetic peptide. (A) Reversed-phase chromatography C18 NST, 5 µm, 250 mm×10 mm column. Mobile phase conditions: H_2_O:ACN:TFA (95∶05:0.1, v:v:v) for 5 min, than a linear gradient to H_2_O:ACN:TFA (05∶95:0.1, v:v:v) in 60 min at a flow rate of 2.5 mL.min^−1^ and detection at 216 nm. Arrow indicate the main fraction containing *Pa-*MAP with retention time of 33.8 min. (B) MALDI Mass spectrometry analysis of *Pa-*MAP; monoisotopic mass [M+H^+^] = 2212.37. Inner squares represent the Na^+^ and K^+^ ion adducts.

*Pa-*MAP was synthesized following the design for two 11-residue repeating segments from HPLC-8, with the following sequence: H-His-Thr-Ala-Ser-Asp-Ala-Ala-Ala-Ala-Ala-Ala-Leu-Thr-Ala-Ala-Asn-Ala-Ala-Ala-Ala-Ala-Ala-Ala-Ser-Met-Ala-NH_2_. *Pa-*MAP was purified by semi-preparative reversed-phase chromatography with linear acetonitrile gradient of 5 to 95% ACN over water, with 0.1% TFA (Figure 1A). The chromatographic profile shows a major product being eluted with 48% of acetonitrile with minor contaminants with a retention time of 33.8 min. MALDI-ToF evaluation showed an ion with 2212.4 m/z, corresponding to the calculated value for the peptide sequence, with above 95% of purity for the isolated product (Figure 1B). Furthermore, K^+^ and Na^+^ adducts were also observed. All further bioassays were performed using this purified fraction, now named *Pa-*MAP.

### Antibacterial Tests

The microdilution assays were performed in order to determine the ability of the *Pa-*MAP to reduce bacterial growth. *Pa-*MAP was efficient in controlling *E. coli* growth, presenting a MIC of 30 µM. On the other hand, for *S. aureus*, *Pa-*MAP only presented activity at higher concentrations, showing a MIC greater than 115 µM (Table 1).

**Table 1:** Toxicity and minimum inhibitory concentrations (MIC) of *Pa*-MAP against mammalian cells, tumor cells, fungi (yeast and mycelium), Gram-positive and -negative bacteria and viruses.

Organism	MIC value (µM)
***Mammalian***	
Erythrocytes	nt*
RAW 264.7	nt*
Vero Cells	nt*
***Cancer Cells in Culture*** **	
*CaCo-2*	60
*HCT-116*	>115
*MCF-7*	115
***Fungi***	
*T. mentagrophytes*	115
*T. rubrum*	115
*C. parapsilosis*	>115
***Bacteria***	
*E. coli*	30
*S. aureus*	>115
***Virus*** **	
HSV-1	90
HSV-2	90

* Non-toxic concentration at 115 µM.

** Concentration used for ED_50_ = Effective Dose.

### Antiviral Tests

By using Vero cells, the MNTC was determined as 90 µM for *Pa-*MAP. The activity of the peptide against HSV-1 and HSV-2 was tested through peptide titration until reaching non-cytotoxic concentration (MNTC). *Pa-*MAP caused 82% of HSV-1 inhibition at a concentration of 45 µM and 90% of HSV-2 at 23 µM. Moreover, 94 and 97% of inhibition for both HSV-1 and HSV-2 were observed at 90 µM (Table 1).

### Antifungal Tests

The broth microdilution assay was performed in order to determine the ability of *Pa-*MAP to inhibit the development of mycellar fungi *T. mentagrophytes* and *T. rubrum* as well as the yeast *C. parapsilosis*. Growth inhibition for both *T. mentagrophytes* and *T. rubrum* was observed and the peptide presents a MIC of 115 µM. On the other hand, *C. parapsilosis* incubated with *Pa-*MAP demonstrated a MIC higher than 115 µM (Table 1), showing lower effects of *Pa-*MAP toward the yeast here evaluated.

### Cytotoxic Studies for Tumoral Cells

The effects of *Pa-*MAP against tumor cells in culture were evaluated by using three cell lines: Caco-2 (human epithelial colorectal adenocarcinoma cells), HCT-116 (human colorectal carcinoma cell lines) and MCF-7 (human breast cancer cell). *Pa-*MAP showed activity against all tumor cells, reaching at 63, 31 and 55% inhibition for Caco-2, HCT-116 and MCF-7, respectively; at a concentration of 115 µM. ED_50_ was calculated for Caco-2 and MCF-7, reaching 58 and 110 µM respectively.

### Cytotoxicity Studies

In order to investigate the hemolytic effects of *Pa-*MAP, red blood cells (RBCs) were incubated in phosphate-buffered saline, 50 mM, pH 7.4 (negative control), Triton X-100 (positive control) and also with several peptide dilutions (16, 32, 64, 128 and 256 µg.mL^−1^). *Pa-*MAP did not show any hemolytic effect (Table 1). In addition, no effects were seen of *Pa-*MAP toward the RAW 264.7 (mouse leukemic monocyte macrophage) cell line, which was observed to be viable in maximum peptide concentration assayed.

### Circular Dichroism Analysis of Pa-MAP

**Figure 2:**
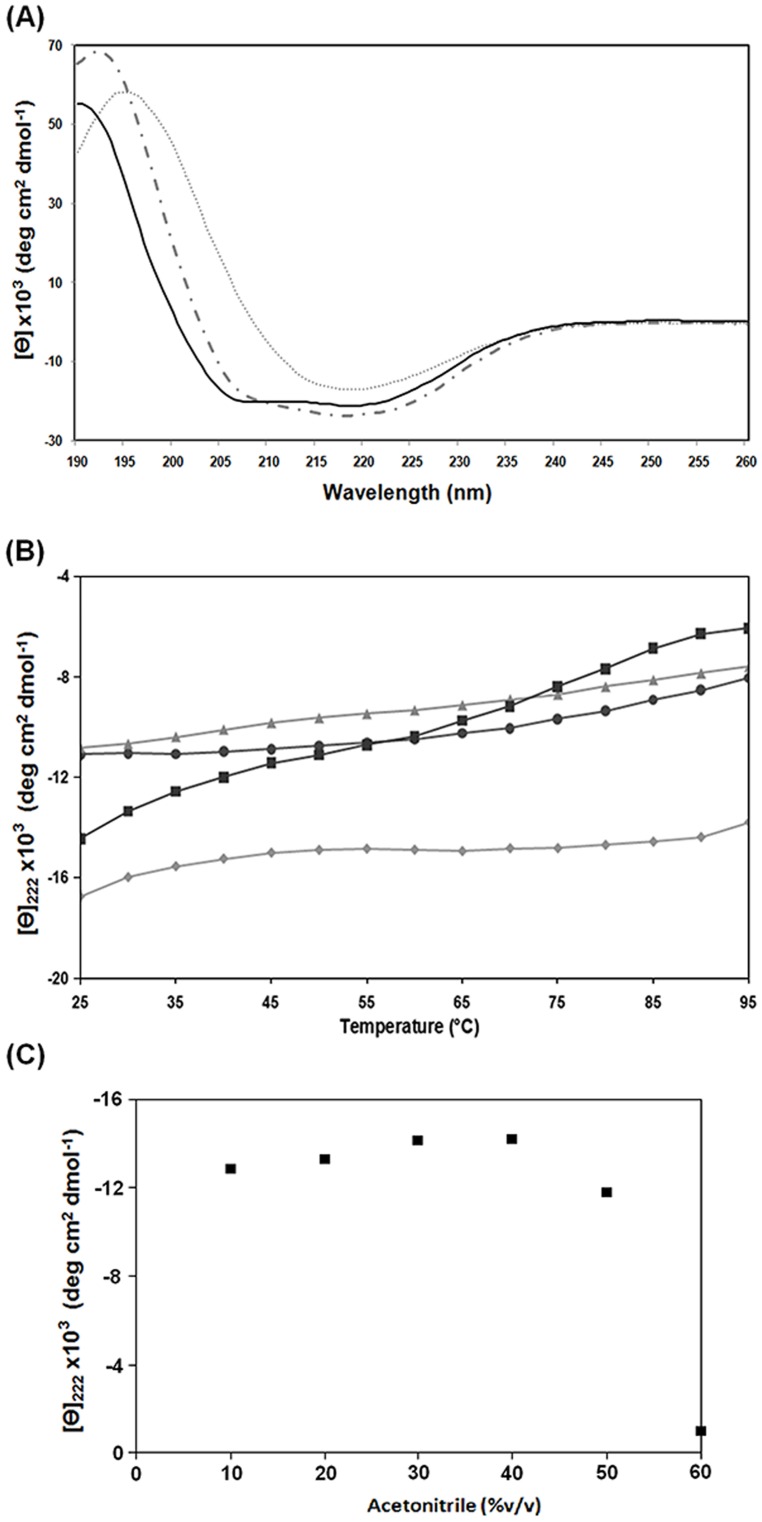
Conformational changes of *Pa-*MAP evaluated by Far-UV circular dichroism in water, TFE and SDS environments. (A) *Pa-*MAP CD spectra in water (dotted line), 28 mM SDS (dotted and dashed line) and 50% (by volume) TFE (continuous line). (B) Thermal and pH denaturation profiles of *Pa-*MAP. The symbols represent ▴ pH 3.0; ♦ pH 7.0; • pH 8.5 and ▪ pH 11.0. (C) Conformational hydrophobic effect of acetonitrile (v:v) on *Pa-*MAP. Molar ellipticity was monitored at 222 nm.

The secondary structure of *Pa*-MAP was investigated using CD spectroscopy in water, SDS 7 and 28 mM, and TFE 50% solutions. CD spectrum in water (pH ∼7.0) showed the presence of a broad negative band around 216−218 nm and positive band ∼198 nm characteristic of the β-sheet structure (Figure 2A). This conformation was observed at several peptide concentrations, starting at 0.05 and progressing to 0.5 mg.mL^−1^ (data not shown). Otherwise, in SDS 28 mM (anionic micelle) the CD spectrum is arrested up to single minimum at ∼218, with a turning point at ∼208 nm, suggesting the presence of both β-sheet and α-helix structures. In the presence of TFE 50% the CD spectrum shape indicated that peptides adopt a defined α-helix secondary structure, marked by two minima around 208 and 222 nm, the positive band at 190 nm and molar ellipticity around zero at 200 nm. In addition, the helical contents were around 55% in aqueous TFE (at 50% by volume) and reached 60% at two different concentrations of SDS (Table 2).

**Table 2:** Secondary structure content of *Pa*-MAP using the method described by Morrisett and coworkers 1973.

Peptide	[θ]_222_	Environment	Fraction helix (%)
*Pa*-MAP	−21912.6	SDS (7.0 mM)	60.9
*Pa*-MAP	−22742.3	SDS (28.0 mM)	63.2
*Pa*-MAP	−20121.0	TFE (50%)	55.9

Thermal stability of the synthetic peptide was also evaluated in water, 2 mM Na-acetate buffer at pH 3.0, 2 mM ammonium bicarbonate buffer at 8.5 and 2 mM glycine-NaOH buffer at 11.0. The thermal unfolding curves in water, at pH 3.0 and 8.5, showed that the secondary structure of the peptide was preserved at temperatures ranging from 25 to 95°C (Figure 2B). In contrast, at pH 11.0 and 65°C a significant conformational loss, reaching approximately 45% of reduction, was observed (Figure 2B). In addition, the effect of acetonitrile was investigated in order to determine if hydrophobic interactions contributed to *Pa*-MAP stability. Aqueous acetonitrile solutions did not disturb the secondary structure up to 40% (by volume) (Figure 2C). However, a significant conformational change in the peptide was observed at 50% and a total loss of the secondary structure at 60%, with a consequent higher decrease in the dichroic signal.

### Molecular Modeling of Pa-MAP

**Figure 3:**
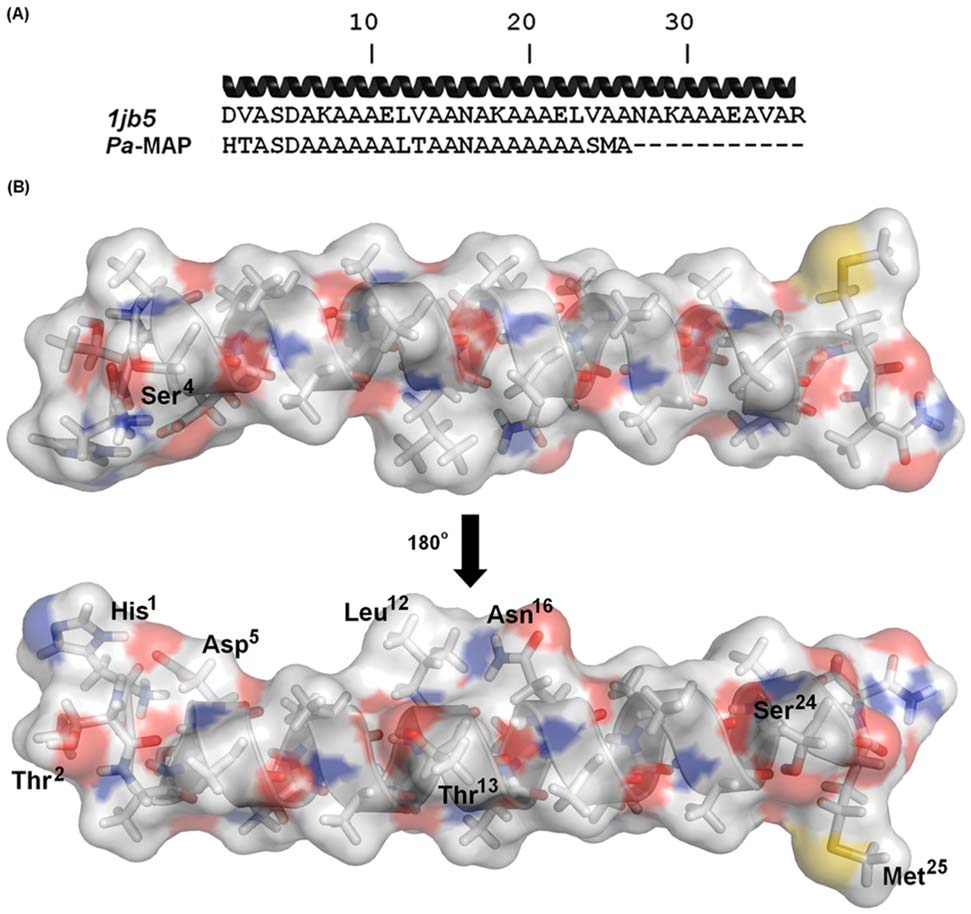
Theoretical tridimensional structure of *Pa-*MAP. (A) Multiple alignment of *Pa-*MAP and the template (1jb5) used for tridimensional model construction. The black helix at the top of alignment represents the template secondary structure. (B) Tridimensional model of *Pa-*MAP constructed by Modeller 9.v.8 with the electrostatic surface calculated with APBS. Blue surfaces represent basic charges (the amino terminus) and red surfaces represent the acidic ones. Amino acid residues possibly involved in the interaction with multiple membrane-targets were labeled and highlighted.

The primary sequence of HPLC-6 hydrophobic antifreeze peptide from *P. americanus* showed 62% of identity with *Pa-*MAP (Figure 3A) and its NMR tridimensional structure (pdb 1j5b) was used as template for molecular modeling [38]. The *Pa-*MAP model shows α-helical conformation (Figure 3B); this has also been observed in data obtained through prediction servers, such as PredictProtein and SOPMA, which presented 92 and 88% of the amino acid residues favoring this conformation [39], [40]. The Procheck summary of *Pa-*MAP showed that 95.8% of amino acid residues are located in the most favorable regions, and only 4.2% are in the region allowed for helix formation. Structural differences between the template structures and predicted three-dimensional structure of the peptide model were calculated by superimposing Cα traces and backbone atoms onto the template structures. The RMSD values between the experimental and theoretical models were 0.5 Å. The RMSD value and low variability among the structural templates and the model structure reflect conservation in most regions and emphasize a similar folding pattern throughout this peptide.

### Molecular Dynamics of Pa-MAP

**Figure 4:**
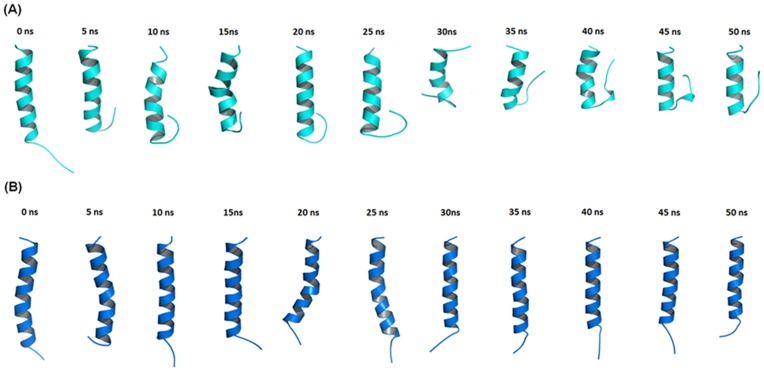
Molecular dynamics evaluation of *Pa-*MAP. Structural snapshots along the 50 ns MD trajectory of *Pa-*MAP in water (light blue) (A) and TFE at 50% by volume solution (dark blue) (B) during the run. Structures are represented as cartoon and further visualized with PyMol http://pymol.sourceforge.net/. The amino terminus of the peptide is always in the bottom (top).

**Figure 5:**
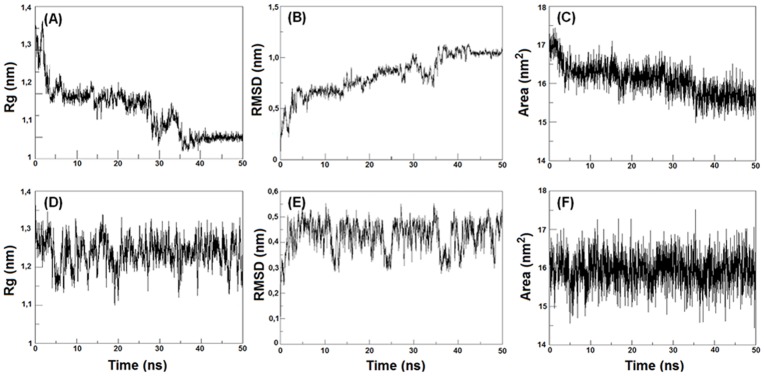
Pa-MAP molecular dynamics simulations. Simulations analysis of conformational stability were performed in water (upper panels) and 50% aqueous TFE (down panels) solution. Physical-chemical parameters such as radius of gyration (Rg) (A and D), root mean square deviation (RMSD) (B and E) and accessibility area for solvent (Area) (C and F) are plotted during 50 ns of simulation.

In addition, *in silico* studies of *Pa-*MAP conformational dynamics in water and TFE solution were carried out. Figure 4 presents an overall view of the dynamic simulation for 50 ns with different snapshots of 5 ns runs conducted in water (A) and in the presence of TFE (B). In the simulation with water (Figure 4A) it was evident that the *Pa-*MAP underwent conformational modification after 30 ns of the run and the long helix was partially unfolded. Otherwise, in the simulation with TFE (Figure 4B) no modification was observed, and the peptide remained in α-helical conformation during the entire simulation. The α-helix stability observed in MD simulations is in agreement with the CD signal measured. The dynamic simulation was used to analyze some physical-chemical parameters, such as the radius of gyration (Rg), root mean square deviation (RMSD) and accessibility area for solvent (Area) for 50 ns, in two environments (water and TFE solution). These parameters were used to furnish data for the different conformations adopted in two different environments. The modifications observed in Rgs for Pa-MAP in water and TFE were 0.34 and 0 nm respectively; and this might be clearly observed after 30 ns of simulation in water indicated by a sloping negative line (Figure 5A). Nevertheless, in the TFE environment, no modification was observed during the simulation (Figure 5D). The change on RMSD values for the initial (0 ns) and final model (50 ns) for both simulation analyses were 0.6 and 0 nm, respectively (Figures 5B and 5E). The results demonstrated only a few modifications after 50 ns of simulation for this peptide and demonstrated that in water and TFE at 20°C, *Pa-*MAP kept the α-helix content at 42 and 90%, respectively (Figures 4A and 4B). The solvent accessible area presented variation values of 2.1 and 0 nm^2^, which are represented by a sloping negative line (Figures 5C and 5F). Data provided by CD demonstrated that *Pa-*MAP presented a partial α-helical structure, which was dynamically most favorable after 35 ns of dynamic simulation.

## Discussion

The emerging incidence of antimicrobial resistance mechanisms developed by microbial pathogens is currently a serious worldwide threat to public health. It is particularly dangerous for immune-compromised patients, and those undergoing anticancer chemotherapy or therapy after organ transplants [41]. Opportunistic pathogens such as bacteria, viruses and fungi can invade various tissues and cause systemic infections, which are considered life-threatening to the patient [42]. In addition, the infectious diseases caused by antibiotic-resistant microorganisms have contributed to making the situation worse, especially for those patients whose treatment with currently available drugs has become less efficient [43], [44].

Due to all these facts, peptides with multiple activities have been extremely attractive for their efficient control of natural resistance episodes in microorganisms, mainly because they show low toxic effects on mammalian cells [45]. These molecules can be obtained from a wide variety of sources, including microorganisms, plants, amphibians, insects, mollusks and fish, presenting a broad range of activity [2]. Here, an alanine-rich peptide was designed from a polar fish, *P. americanus*, with two repeat antifreeze motifs, as previously reported by Holmberg *et al.* [14], and clear deleterious activities toward bacteria, fungi, viruses and cancer cells were observed. One of the promiscuous peptide classes is the defensins, which are small, basic and cysteine-rich peptides found in numerous organisms including plants, fungi and humans. Defensins are generally active against a broad spectrum of organisms, such as Gram-positive and Gram-negative bacteria, viruses, fungi and nematodes [46]. Additionally, cyclotides, a family of widely studied plant-derived promiscuous polypeptides also present numerous activities, including antimicrobial, cytotoxic, insecticidal, uterotonic, antiviral, neurotensin antagonism, hemolytic and anthelmintic ones [47]. Recently, a disulphide-free plant peptide from *Cocos nucifera*, named *Cn*-AMP1, was characterized as a promiscuous peptide presenting functions that include antibacterial, antifungal, antitumor and immuno-stimulatory activity [48], [49]. Animal peptides such as magainin (*Xenopus laevis*), mastoporan (*Vespa simillima*), fowlicidin (*Gallus gallus*) and LL-37 (*Homo sapiens*) have also demonstrated multiple functions, including antimicrobial, anticancer, antiviral, insecticidal and hemolytic ones [50]–[53]. In addition, antimicrobial peptides from teleost fish have been linked to multifunctional activities. Among them can be cited pardaxin (*Pardachirus marmoratus*), hepcidin (*Oreochromis mossambicus*), epinecidin (*Epinephelus coioides*), piscidin (*Morone chrysops*), misgurin (*Misgurnus anguillicaudatus*), NRC peptides (*Pleuronectes americanus*), myxinidin (*Myxine glutinosa*) and CodCath (*Gadus morhua*) [53]–[60].

Firstly, to demonstrate the varied effects of the studied peptide, *Pa-*MAP was assayed against multiple infectious pathogens. Most bactericidal activities have been related to cationic residues in the literature [9], [61]. Despite the fact that arginine and lysine cationic residues seem to have an important role for antimicrobial activity, *Pa*-MAP is devoid of these residues, presenting mostly hydrophobic amino acid residues with one histidine and one aspartic acid residue located in the N-termini region. To probe if the side chain of this His^1^ residue could be protonated at pH 7.0, the pH at which antimicrobial assays were performed, its pKa value was determined by potentiometric titration and was shown to be 6.0, in accordance with the value expected for the free amino acid or for histidine side chains exposed to water. This indicates that antimicrobial activity could be driven mostly by hydrophobic interactions (Figure S1). Furthermore, *Pa-*MAP showed a higher activity toward Gram-negative (MIC = 30 µM) bacteria when compared to Gram-positive (MIC >115 µM) pathogens.

*Pa-*MAP also showed deleterious activity against mycelium and yeast fungi belonging to *Ascomycota* phylum. With this amidated peptide, the polar amino acid residues Thr^2^, Ser^4^, Asp^5^, Thr^13^, Asn^16^ and Ser^24^ create a polar environment for peptide interaction, despite a possible eletrostatic repulsion caused by Asp^5^ residue. Similar compositions were observed in an antifungal peptide from *Trapa natans* fruits with inhibitory effects on *Candida tropicalis* biofilm formation [62]. In addition, our results were similar to those found for antifungal peptides deposited in APD, which demonstrated a GRAVY index range of −0.900 to 1.505 [63], [64] reinforcing the idea that hydrophobic interactions promoted by the multiple alanine residues and by Met^24^ are responsible for the second step of interaction with the lipid hydrocarbon backbone.

*Pa-*MAP also presented antiviral activity. This is possibly due to the presence of hydrophobic amino acid residues, which might interact with the viral envelope and also with phospholipids encountered on the viral surface [65], [66]. Previous descriptions showed various antiviral peptides that presented directly proportional activity in relation to the hydrophobic ratio, suggesting that hydrophobic and aromatic residues are also important for antiviral activity. Lee and coworkers [66] reported that the increase in the hydrophobic ratio for cecropin A-magainin 2 hybrid peptide analogues caused a dramatic increase in virus-cell fusion inhibitory activity against HIV-1 virus. In summary, it seems that different residues might be involved in different functions, especially when the activities against multiple infectious pathogens were evaluated.

Another important concern for peptide pharmacy descriptions consists of evaluating deleterious activities toward mammalian cells. These challenges can be done in two different directions: one against tumor cells to develop anticancer drugs and the other against healthy cells to probe peptide safety. *Pa-*MAP showed clear activity toward different tumor cell lines in culture. Its antitumor activity, like its antiviral ones, could be related to a higher hydrophobicity ratio (73%) and to the presence of Thr^2^, Leu^12^, Thr^13^ and Met^25^. These residues were commonly encountered in anticancer peptides, as observed for pardaxin 1, a multifunctional peptide from *Pardachirus marmoratus,* and in a promiscuous peptide from *Epinephelus coioides* [67]. Another recent study demonstrated that antitumoral activity improved as hydrophobicity increased for a peptide with a length of 26 residues and an α-helical structure was derived from a small replication protein (RepA) from *E. coli* [68], [69]. A more detailed analysis of hydropathicity was carried out using the GRAVY index [33], and it was observed that anticancer/antitumor peptides deposited in the antimicrobial database (APD) [70] mostly presented a GRAVY index around −0.823 to 1.3, which corroborates our results, since *Pa-*MAP presented a GRAVY index of 0.888 [71], [72]. Otherwise, *Pa-*MAP showed no toxic effects against human erythrocytes, RAW 264.7 and Vero cells at the maximum concentration (115 µM) utilized for all assays (Table 1). A multifunctional peptide from the Chinese scorpion *Mesobuthus martensii* Karsch, named BmKbpp, also showed hemolytic activity around 40% at 50 µM [73]. This suggests that *Pa-*MAP may be a candidate for use as a model for rational design of antibiotic peptides used in the treatment of human diseases caused by pathogenic microorganisms. It seems that the key to toxicity to mammalian cells might be related to membrane composition. Erythrocyte membranes are composed of phospholipids such as phosphatidylcholine (PC) and sphingomyelin (SM) [74], along with the presence of cholesterol. [75] Moreover, the absence of activity toward mammalian cells could be related to the lack of arginines and lysines. For melittin, a promiscuous peptide from *Apis mellifera* [76], it was demonstrated that mutations of arginine and lysine residues changed the total activity, with a major effect on toxicity to mammalian cells, reducing 8-fold the activity of the peptide after modification [77].

After biochemical characterization *Pa-*MAP was also analyzed for its biophysical parameters under different conditions. *Pa*-MAP was analyzed by circular dichroism, molecular modeling and dynamic simulation, showing that in a hydrophilic environment the conformation is dynamically more unstable at the N- and C-terminals. In contrast, the peptide structures were stabilized in all portions in hydrophobic environments. Many NMR studies with antimicrobial peptides demonstrated that a helical structure is favored in micellar media. MSI-594, a magainin variation of an antimicrobial peptide, possesses a parallel orientation in LPS micelles and, interestingly, the conformation in dodecylphosphocholine (DPC) micelles showed a straight ←helix without any long-range packing, as observed in LPS [78]. Studies with temporin analogues, a representative frog-derived AMP and urechistachykinin peptide from the peripheral nervous system of invertebrates such as *Urechis unicinctus* [79], showed that the native peptides presented α-helical conformation in the SDS environment. The addition of TFE also demonstrated the formation of α-helical structure in a similar manner to that observed in other helical peptides in TFE/water mixtures [80].

In order to determine if hydrophobic interactions contributed to the stability of the *Pa-*MAP helix, several acetonitrile concentrations (v:v) were assayed. This solvent does not significantly disturb the hydrogen bond stabilizing the helix conformation, as also observed in studies of hydrophobic effects on micelles and biological membrane formation (SDS and TFE solutions). However, some destabilizing effect occurred at high acetonitrile concentrations since the solvent has moderate capability either as hydrogen-bond donor or as hydrogen-bond acceptor [81]. Figure 2C shows that the main effect of acetonitrile was to destabilize the helix above 60% concentration.

*Pa*-MAP folding in water and TFE was also studied by molecular dynamics using two different initial conformations for the peptide modeled by molecular modeling, thermalized and energy minimization in aqueous phase (A1) and TFE phase (A2). In the A1 phase there is one central helical region in the peptide in which the first three (N-termini), and the last five (C-termini) amino acid residues are nearly unstructured and appear as coils or bends. This extension of the secondary structure is in agreement with the CD data in aqueous solutions. Figure 4A shows the A1 simulation at different moments: one can see that the peptide stabilized at 30 ns and presented N- and C-termini flexibility with around 30% of helical structure unfolding, when comparing the initial (0 ns) and final (50 ns) models in a thermalized system. Stability was also observed through several parameters, including Rg, RMSD and area, and it was demonstrated that in a hydrophilic environment *Pa-*MAP presents greater instability at the N- and C-terminals. Nevertheless, the conformational predominance was clearly a α-helix whose stability is maintained far from the terminals. Similar results were obtained by Ding and coworkers [82], who evaluated a synthetic alanine-rich peptide, known as AK17, and its analogues AK10G and AK9P, through CD and molecular dynamics. These peptides presented a radius of gyration of 1.05, 0.89 and 0.92 nm, values that are similar to those observed for *Pa-*MAP at 1.2 nm. The RMSD values obtained for *Pa-*MAP (0.6 nm) are also in accordance with data obtained in a pure water dynamic simulation for melitin at 0.8 nm [83]. In the A2 phase, *Pa-*MAP was assayed in the TFE medium, which induces the formation of a α-helical structure also observed for *in vitro* experiments. Similar data were found for helical synthetic peptides resembling most α-helices found in the native proteins in TFE/water mixtures [80]. *Pa-*MAP reaches 90% extension in an α-helical conformation in the presence of TFE, being stabilized after 5 ns and remaining so for the 50 ns of simulation. For the A2 system, the obtained result was similar to the results of atomistic simulations in TFE/water mixtures related to C-terminal fragments named Aβ42, obtained from amyloid β-protein (Aβ), a key neurotoxin in Alzheimer’s disease [84], [85]. Similar results were demonstrated by Soufian and coworkers [86], who observed the structural stability of aurein 1.2, an amphipathic peptide with antibacterial and anticancer activity, and its retro analog in TFE/water mixture, where RMSD, Rg and area were evaluated by molecular dynamics.

In summary, the alanine-rich α-helix, together with the hydrophobic Leu^12^ and Met^25^ amino acid residues, and the presence of polar and negatively charged residue jointly with amidated C-terminal, convert *Pa-*MAP into an amphipathic molecule that appears to have the ability to interact in several membrane compositions. Additionally, data reported here suggest that *Pa-*MAP in a hydrophilic environment possesses unstable N- and C-terminals, which favor anchorage and further interactions with different acid phospholipids, probably mainly at the charged amino group. In the hydrophobic environment of phospholipid membranes, it was hypothesized that the folding of *Pa-*MAP is guided by hydrophobic interactions to achieve more stable structural conditions. Moreover, this structural flexibility, which allows a switch from helix to coil and vice-versa, according to the medium, enables this peptide to act against different cells, leading us to believe that the multiple actions could be driven by a mixture of composition, three-dimensional structure and solvation.

### Conclusions

The understanding of *Pa-*MAP structural stability and conformational preference at the molecular level in a hydrophobic environment may lead to advances in drug design and therapy. Because of the ever-increasing number of drug-resistant bacteria, healthcare worldwide is facing a serious challenge and there is an urgent need for novel compounds to treat diseases. Host-defense peptides have high potential to become the next generation of bioactive compounds. Understanding the structure-function correlations of these multifunctional peptides could be critical for developing nontoxic therapeutic biotechnological tools. In the literature the majority of reports have demonstrated that antimicrobial activity could be associated with two main physical-chemical parameters: charges and hydrophobicity. This study demonstrated that both properties are important, but are only part of the process. *Pa-*MAP demonstrated environment-dependent folding according to *in vitro* and *in silico* assays, presenting greater conformational stability in hydrophobic media. In summary, deeper understanding of structure-functional relations could help researchers to develop more efficient and safer peptide drugs, with higher affinities against pathogens and cancer cells in comparison to other mammalian cells. Moreover, the understanding of how environmental conditions such as pH, temperature and hydrophobicity can modulate peptide activities due to smooth structural fluctuations could also be important, allowing the development of multidrugs that act at different moments and under varying conditions.

## Supporting Information

Figure S1 **Titration curve of** ***Pa*** **-MAP in with sodium hydroxide.** Experiment was performed with 0.27 mM of Pa-*MAP* titrated with 1 mM sodium hydroxide.
_(TIF)_
