# Supplementary figures and images for: Structural and Functional Characterization of a Multifunctional Alanine-Rich Peptide Analogue from Pleuronectes americanus
Source: PLoS One. 2012 Oct 8;7(10):e47047. doi: 10.1371/journal.pone.0047047 (PMC3466273; doi:10.1371/journal.pone.0047047)

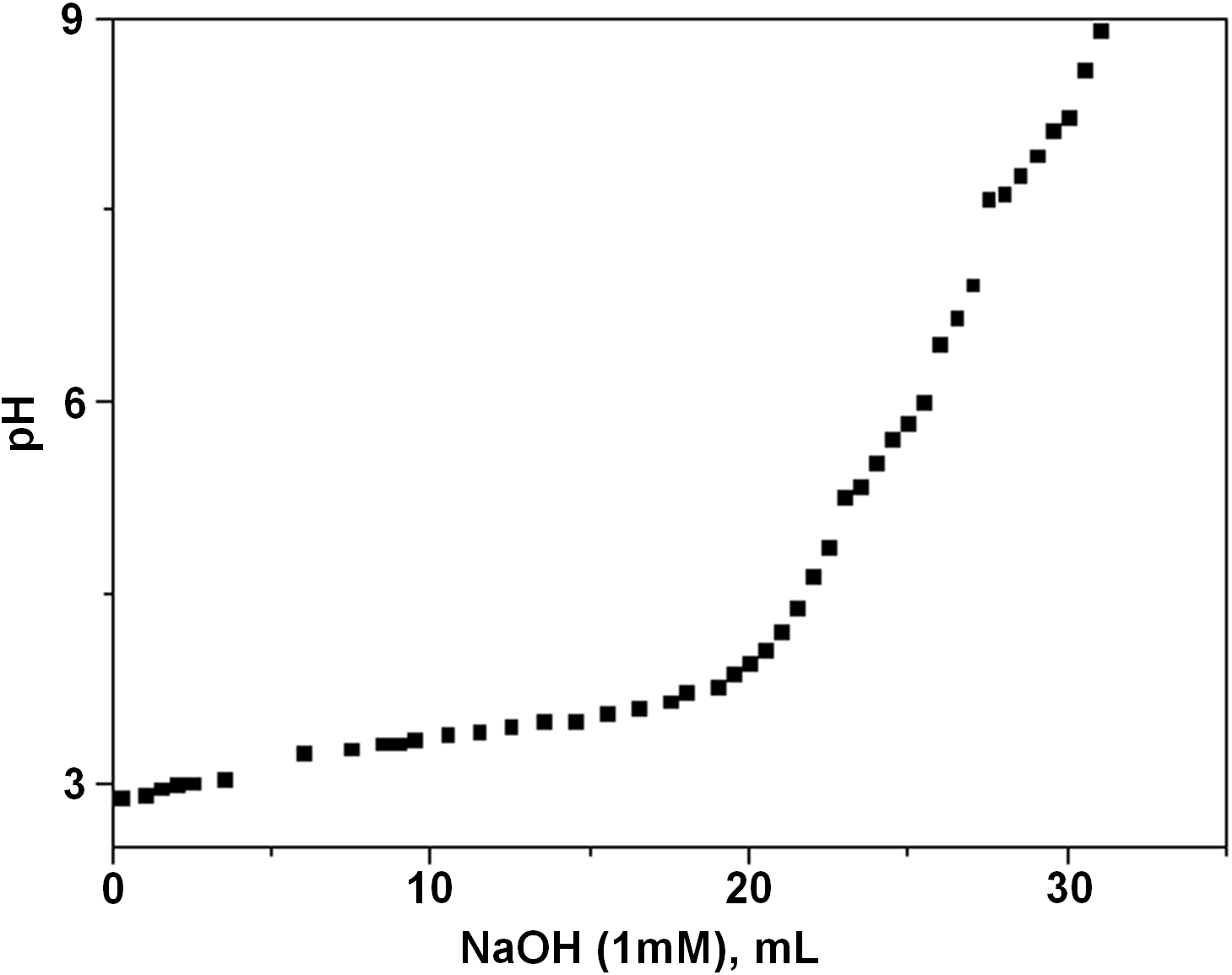

Supplement: Figure S1 — Titration curve of Pa -MAP in with sodium hydroxide. Experiment was performed with 0.27 mM of Pa-MAP titrated with 1 mM sodium hydroxide. (TIF) [file pone.0047047.s001.tif]
